# Heterogeneity of ictal firing during generalized seizures in the awake cortex

**DOI:** 10.1093/cercor/bhag049

**Published:** 2026-04-30

**Authors:** Péter Sere, Olivér Nagy, Nikolett Zsigri, Vincenzo Crunelli, Magor L Lőrincz

**Affiliations:** Department of Physiology, Faculty of Medicine, University of Szeged, 10 Dóm sqr, Szeged 6720, Hungary; Department of Physiology, Faculty of Medicine, University of Szeged, 10 Dóm sqr, Szeged 6720, Hungary; Department of Physiology, Faculty of Medicine, University of Szeged, 10 Dóm sqr, Szeged 6720, Hungary; School of Biosciences, Cardiff University, Museum Avenue, CF10 3AX Cardiff, United Kingdom; Department of Neuroscience and Pharmacology, Faculty of Medicine, Lisbon University, Avenida Professor Egas Moniz, 1649-028 Lisbon, Portugal; Department of Physiology, Faculty of Medicine, University of Szeged, 10 Dóm sqr, Szeged 6720, Hungary

**Keywords:** brain states, cell-types, inhibition, neocortex, spike and wave discharges

## Abstract

Cortico-thalamo-cortical oscillations are central to both normal and pathological brain activities and emerge from complex cortical and thalamic interactions. However, the specific activity of identified cortical neurons during the paroxysmal oscillations associated with absence seizures (ASs) in awake animals remains underexplored. The dominant narrative suggests that seizures indiscriminately disrupt cortical activity through uniform increases in neuronal excitability; however, direct evidence for such homogeneous recruitment at the single-neuron level is lacking. Here, we recorded single units from pyramidal neurons and different interneuron subtypes in the neocortex of two validated rodent models of absence epilepsy under awake, behaving conditions. We find that neurons maintain their firing rank order across interictal and ictal states, regardless of whether their ictal firing rate increases, decreases, or remains stable compared to the interictal phase. Rather than a random cortical takeover, ictal activity represents a scalable modulation of pre-existing network states. These results challenge the generalized hyperexcitability model and highlight the structured, heterogeneous nature of cortical activity during ASs, with implications for mechanistic understanding and targeted therapies.

## Introduction

Epilepsy is a debilitating neurological disorder affecting ~1% of the global population, leading to diminished quality of life, comorbidities, and mortality (Fisher et al. 2014). Despite extensive research using diverse *in vivo* and *in vitro* models, the cellular mechanisms underlying seizure generation, propagation and termination remain incompletely understood. The prevailing view portrays cortical activity during seizures as homogeneous and hypersynchronous, driven by disrupted excitation–inhibition balance (Engel 1996). However, mounting evidence indicates that neuronal firing is far more heterogeneous in both experimental models (Matsumoto and Marsan 1964; Sawa et al. 1968; Bower and Buckmaster 2008; Meyer et al. 2018; McCafferty et al. 2023) and human patients (Truccolo et al. 2011). Indeed, human single-unit studies during both interictal events and seizures reveal marked heterogeneity: the firing rate of only a subset of neurons is modulated during interictal discharges with diverse patterns—including pre-event increases and decreases near the focus—and seizure initiation/spread is characterized by mixed rate changes rather than monolithic recruitment (Keller et al. 2010; Truccolo et al. 2011).

The cerebral cortex hosts a diverse population of neurons that differ in morphology (Szentágothai 1978), electrophysiology (McCormick et al. 1985), and neurochemistry (Lee et al. 2010). Recent transcriptomic studies have revealed even greater diversity (Gouwens et al. 2020), with neuronal identity linked more closely to brain state than to morphology or intrinsic physiology (Bugeon et al. 2022). These findings suggest that structured neuronal heterogeneity might be a defining feature of cortical organization. Indeed, during physiological oscillations such as sleep slow waves (Steriade et al. 1993), gamma (Varga et al. 2014), alpha (Lorincz et al. 2009), or sleep spindle (Gardner et al. 2013) oscillations neurons exhibit variable firing across cycles. Thus, synchronized network rhythms can coexist with pronounced single-cell variability—implying that heterogeneity is not noise, but an organized property of cortical dynamics.

Paroxysmal activity, characterized by excessive synchrony and large-amplitude electroencephalogram (EEG) deflections, is generally attributed to altered excitation/inhibition balance or hyperexcitability (Lőrincz et al. 2024). Work *in vitro* and anesthetized preparations suggest that cortical neurons discharge synchronously during such events (Ziburkus et al. 2006). Yet, studies in awake behaving animals have revealed a different picture: cortical (Meyer et al. 2018; McCafferty et al. 2023), thalamic (McCafferty et al. 2018, 2023), and striatal (Miyamoto et al. 2019) neurons show variable and less entrained activity. This discrepancy suggests that pharmacological manipulations, such as anesthesia may artificially enhance synchrony, masking the intrinsic heterogeneity present during seizures under natural conditions.

Thus, the neuronal dynamics underlying seizure generation, propagation, and termination in the awake cortex remain poorly understood. The dominant narrative suggests that seizures overtake cortical networks in a chaotic and indiscriminate manner, but the supportive evidence is lacking. If physiological brain rhythms already express structured heterogeneity, could pathological oscillations such as spike-and-wave discharges (SWDs) of absence seizures instead reflect a scalable modulation of pre-existing network states rather than a random cortical takeover? Building on ensemble studies that documented mixed ictal rate changes in cortex and thalamus in awake rodents (McCafferty et al. 2018, 2023; Meyer et al. 2018; Miyamoto et al. 2019), we provide cell-type–resolved, single-neuron recordings from identified pyramidal, regular-spiking, and fast-spiking neurons in awake cortex across two validated models of absence epilepsy. We find that neurons maintain their firing rank order across interictal and ictal states, regardless of whether their ictal firing rate increases, decreases, or remains stable compared to the interictal state. Moreover, the magnitude of ictal modulation inversely scales with baseline rate and covaries with spike-and-wave entrainment, revealing a principled structure to this heterogeneity. Thus, SWDs emerge as a scalable reweighting of cortical dynamics—extending prior work with cell-type and cross-species resolution—and challenge the hyperexcitability trope, indicating a structured, hierarchical modulation of cortical networks.

## Materials and methods

All procedures complied with the European Communities Council Directives of 1986 (86/609/EEC) and 2003 (2003/65/CE) for animal research and were approved by the Ethics Committee of the University of Szeged. We used Stargazer mice (*n* = 5, 2 females; 3 to 7 mo of age), a well-established monogenic model of absence epilepsy (Noebels et al. 1990), and Genetic Absence Epilepsy Rats from Strasbourg (GAERS) (*n* = 7, 4 females; 3 to 7 mo), a validated polygenic model (Depaulis et al. 2016). Animals were maintained on a 12:12 h light:dark cycle with ad libitum access to food and water. They were group-housed prior to surgery and singly housed thereafter to protect head implants.

Habituation and recording procedures followed our previous work (Molnár et al. 2021; McCafferty et al. 2023). Briefly, anesthesia was induced with 4% isoflurane in oxygen (2 L/min) in an induction chamber and maintained via a facemask with active scavenging. During surgery, isoflurane was gradually reduced to 1.2%–1.8% in oxygen (1 L/min), with depth monitored by respiratory rate and pedal reflex. Animals were mounted in a stereotaxic frame (Model 902, David Kopf Instruments, USA), the skull was exposed, and a stainless-steel head post was cemented over the frontal suture using dental acrylic (Pattern Resin LS, GC America, USA). Craniotomy sites were marked at the following coordinates: rat S1 (primary somatosensory cortex): 0.0 mm anteroposterior, 2.5 mm lateral to bregma; mouse M1 (primary motor cortex): −1.8 mm anteroposterior, 2.0 mm lateral to bregma.

For postoperative care, animals received carprofen (Rimadyl; 5 mg/kg, i.p.; Pfizer, USA) and gentamicin (0.1 mg/kg, i.m.). After at least 5 days of recovery, animals were handled daily for 7 to 21 days to reduce stress during head fixation, with fixation duration increased gradually.

On recording days, small craniotomies (0.8 to 1 mm) were made under isoflurane (Forane, AbbVie, USA; 1.0% to 1.5% in O₂ at 1 L/min) at the marked positions, leaving the dura intact. Mineral oil was applied to prevent dural dehydration. Animals were then transferred to the in vivo electrophysiology setup; head posts were secured in a custom holder, and recording began ≥40 min after full recovery from anesthesia.

Electrophysiology. Single-unit extracellular recordings were obtained in rat S1 and mouse M1 using either glass micropipettes filled with 0.5 M NaCl (impedance 3 to 8 MΩ) except 6 mouse pyramidal neurons recorded with single-shank, 32-channel silicon probes (NeuroNexus, USA). A proximal (≈0.3 mm) wire electrode (50-μm diameter polyimide-insulated tungsten wire, California Fine Wire, Grover Beach, CA, USA) positioned in infragranular layers was used to record the local field potential (LFP). Glass-electrode signals were preamplified with Axon HS-9A headstages (Molecular Devices), amplified with an Axoclamp 900A (Molecular Devices, USA), and filtered at 0.3 to 6 kHz for single units. LFPs (0.1 to 200 Hz) were recorded with an AC amplifier (Supertech, Hungary; gain 1,000×). Signals were digitized at 30 kHz using a CED Power1401-3 A/D converter (CED, UK) and Spike2 software. Silicon-probe data were amplified, filtered, and digitized with an Intan RHD2000 board (Intan Technologies) at 30 kHz. Offline analysis was performed in Spike2 (CED, UK) and OriginPro 8.5 (Microcal, USA); figures were prepared in OriginPro 8.5. Data are reported as mean ± SD unless noted.

Seizure times were first identified by amplitude threshold crossings in the LFP and then confirmed by the presence of spike-and-wave discharges (SWDs) at 6 to 8 Hz with a minimum duration of ≥3 s. Interictal periods were defined as segments without detected spike–wave discharges (SWDs) based on the SWD detection channel, excluding peri-ictal windows (±2 s around SWD onset and offset) and epochs containing movement artifacts. Recordings were obtained in awake, head-restrained animals. To avoid contamination by non-awake brain states, segments showing sustained slow waves, prominent continuous 5 to 9 Hz rhythmic activity consistent with drowsiness, or REM-like theta-dominant epochs were excluded based on LFP spectral content and visual inspection. No interictal epileptiform discharges were detected in the analyzed interictal segments. For spectral characterization, LFP signals were z-score normalized per recording using interictal baseline and power spectral density was estimated with Welch’s method; relative band power was computed in standard frequency bands (delta: 1 to 4, theta: 5 to 7, beta: 13 to 30, low gamma: 30 to 50 and high gamma: 50 to 80 Hz).

The modulation index (MI) was computed as


$$ \mathrm{MI}=\frac{F{R}_{\mathrm{ictal}}-F{R}_{\mathrm{interictal}}}{F{R}_{\mathrm{ictal}}+F{R}_{\mathrm{interictal}}} $$

such that MI = +1 denotes maximal enhancement and MI = −1 denotes maximal suppression of firing during the interictal→ictal transition. For each neuron and state (interictal, ictal), spiking regularity was quantified as the coefficient of variation of interspike intervals: CV = σISI/μISI. The entrainment index (EI) was defined as the fraction of spikes occurring while the SWD “spike” component of each cycle exceeded 50% of its peak amplitude. The rhythmicity index (RI) was defined as the amplitude ratio of the first side-peak to the main peak in the neuron’s ictal spike-train autocorrelogram.

Neuronal entrainment to physiological rhythms during interictal activity was analyzed using custom-written scripts in MATLAB (MathWorks, Natick, MA, USA) together with the Circular Statistics Toolbox. To minimize contamination of LFP phase estimates by unit activity, the raw broadband LFP signal was first notch-filtered using an IIR filter to remove line-noise components. Spike-related artifacts were then removed from the broadband signal by excising a ± 2 ms window around each spike and replacing the removed segment using cubic-spline interpolation. The quality of spike-artifact removal was validated using spike-triggered averages, spike-triggered spectrograms, and comparisons of power spectral density before and after artifact removal. After validation, the cleaned signal was resampled to 1250 Hz and band-pass filtered using zero-phase FIR filters (filter order 256, Kaiser window design) in the following frequency ranges: delta, 1 to 4 Hz; beta, 13 to 30 Hz; low gamma, 30 to 50 Hz; and high gamma, 50 to 80 Hz. For each band, the instantaneous phase of the filtered LFP was obtained using the Hilbert transform. Spike times were then referenced to the instantaneous phase of the corresponding band-limited signal. Preferred phase was quantified from the mean resultant vector, computed as the circular mean of spike phases, $\mathrm{mean}\left(\exp \left( i\phi \right)\right)$, and coupling strength was quantified as the length of the mean resultant vector. Statistical significance of phase locking was assessed using the Rayleigh test for non-uniformity of circular data.

When multiple neurons were recorded from the same animal, single-cell measures (eg, firing rate, MI, EI, RI, CV) were first aggregated per animal to avoid pseudo-replication; group comparisons were then performed on these per-animal summaries. For each outcome, we inspected histograms and Q–Q plots of per-animal summaries and tested normality with Shapiro–Wilk. When normality and homoscedasticity were satisfied (Levene’s test), we used parametric tests; otherwise, we used non-parametric alternatives. When comparing two groups we used unpaired t-test or Mann–Whitney U (Wilcoxon rank-sum). When comparing three groups [eg, pyramidal (PYR) vs regular-spiking interneurons (RSI) vs fast-spiking interneurons (FSI)] we used one-way ANOVA. When comparing paired states (interictal vs ictal within animals) the Wilcoxon signed-rank test was used. All tests were two-tailed with α = 0.05. Exact *P*-values are reported. We avoided t-tests when > 2 conditions were compared. Animals were assigned to surgical preparation and recording order without formal randomization; no group allocation was involved beyond species/strain. Seizure detection used predefined thresholds and frequency/duration criteria and was run blind to neuron identity. Spike sorting and unit inclusion were performed with automated criteria followed by an investigator blind to cell-type labels. Given the exploratory nature and technical constraints of awake single-unit recordings, a priori power calculations were not performed; instead, we report effect sizes and confidence intervals for all key comparisons. Neurons were included in the analysis only if they exhibited sufficient interictal and ictal sampling to reliably estimate firing rate, variability, and modulation indices. Recordings with unstable unit isolation, insufficient seizure sampling, or fragmentary interictal periods were excluded prior to analysis. All inclusion and exclusion criteria were applied uniformly and independently of firing-rate changes or seizure modulation.

## Results

**Figure 1:**
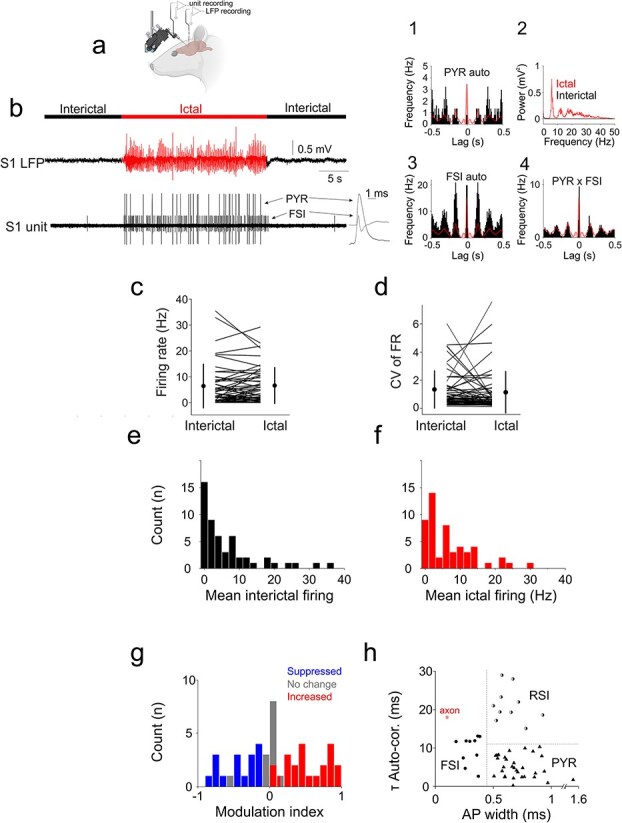
Ictal and interictal firing properties of cortical neurons. (a) Schematics of the experimental design. (b) Example simultaneous recording of two cortical neurons (PYR: Pyramidal neuron; FSI: Fast-spiking interneuron) characterized by ictal entrainment in the S1BF of a GAERS rat. The interictal and ictal periods are indicated with black and red traces, respectively. Averaged action potentials (mean ± s.e.m) are shown on the right on a faster timebase. Ictal autocorrelations of the pyramidal neuron (B1) and fast-spiking interneuron (B3) and their cross-correlation (B4) are shown with the LFP autocorrelation overdrawn in red. Power spectral densities of the LFP interictal (black) and ictal (red) periods are shown in (B2). (c) Mean interictal and ictal firing rates of all recorded neurons. Lines indicate individual neurons; circles indicate mean and standard deviation. (d) Coefficients of variation (CV) of interictal and ictal mean firing rates for all recorded neurons. Lines indicate individual neurons; circles indicate mean and standard deviation. (e) Distribution of mean interictal firing rates. (f) Distribution of mean ictal firing rates. (g) Distribution of modulation indices (MI). Blue bars indicate significant ictal suppression, gray bars indicate no significant change, and red bars indicate significant ictal firing rate increase, respectively. (h) Identification of the recorded neurons. Scatter plot of interictal autocorrelation time constants (τ auto-cor.) vs. action potential width (AP width) clearly separates fast-spiking interneurons (FSI), regular-spiking interneurons (RSI), and pyramidal neurons (PYR).

Because ictal activity is characterized by increased neuronal synchrony that can obscure the distinction of individual units (Huguenard 2019), we performed single-unit recordings in the infragranular layers of the primary somatosensory (S1) barrel cortex of awake, head-restrained GAERS rats and in the primary motor cortex (M1) of Stargazer mice while simultaneously recording the proxymal LFP (Fig.  1a, see Methods). Interictal epochs were defined as SWD-free segments excluding peri-ictal windows and movement artifacts, and no interictal epileptiform discharges were detected in these recordings. To verify that interictal activity reflected physiological awake cortical dynamics rather than sleep-like states, we quantified LFP spectra in clean interictal segments. Interictal spectra were broadband with dominant theta and substantial beta/gamma components (relative band power, mean ± SD: delta 0.048 ± 0.006, theta 0.39 ± 0.02, beta 0.25 ± 0.02, gamma 0.09 ± 0.02; 1 to 80 Hz normalization, based on 38 LFP recordings), consistent with awake cortical activity and distinct from sleep or drowsy states. Periods showing sustained slow waves, REM-like theta dominance, motor artifacts were excluded from analysis. An example interictal versus ictal spectral comparison is shown in Fig.  1B2. From these experiments, we obtained 52 well-isolated neurons (24 from rats, 28 from mice) that exhibited sufficient interictal and ictal epochs for analysis. The use of high signal-to-noise, single-cell recordings ensured that even ictally recorded spikes could be unambiguously attributed to individual neurons (Fig.ure  1a, b, and  h).

Across all recorded neurons, mean interictal (6.61 ± 8.43 Hz) and ictal (6.80 ± 6.95 Hz) firing rates did not differ significantly (*P* = 0.394, Wilcoxon rank-sum test, *n* = 52, Fig.  1c, e, and f). Similarly, the coefficients of variation (CVs) of interictal and ictal firing were comparable (1.32 ± 1.34 vs. 1.12 ± 1.47, *P* = 0.099; Figure  1d). Nevertheless, interictal and ictal firing rates were strongly correlated (ρ = 0.780, *P* = 9.6 × 10^−12^), indicating that neurons retained their relative firing-rate rank across brain states. The relationship between interictal and ictal firing rates was highly stable across neurons (r = 0.86, *P* = 6.8 × 10^−16^). A split-half analysis yielded nearly identical correlations in two independent, randomly selected subsets of cells (r = 0.85 and 0.87), confirming that the preserved firing structure was not driven by a small subgroup but reflected a robust population-level property.

To quantify cell-specific changes, we calculated a modulation index (MI; −1 = maximal suppression, +1 = maximal increase, 0 = no change, Fig.  1g). The MI distribution revealed pronounced heterogeneity (Fig.  1g), with approximately three-quarters of neurons (39/52, 75%) showing significant modulation between interictal and ictal periods (*P* < 0.05, Wilcoxon signed-rank test for individual neurons). Among these, 22 neurons (42%) increased their firing, 17 (33%) decreased it, and 13 (25%) showed no significant rate change (Fig.  1g). The absolute MI was negatively correlated with baseline interictal firing (r = −0.459, *P* = 6.3 × 10^−4^), suggesting that neurons with higher baseline activity were less likely to undergo strong ictal modulation. The MI did not differ significantly between GAERS rats and Stargazer mice (*P* = 0.072), indicating comparable ictal firing modulation across models. Baseline interictal firing rates showed a modest species difference (*P* = 0.042), but this did not translate into differences in ictal modulation strength. No significant differences in modulation index or baseline firing rate were observed between male and female animals (MI: *P* = 0.53; firing rate: *P* = 0.17).

Neurons were classified as PYR, RSI, or FSI based on spike waveform width and autocorrelation time constant (τ) following established criteria (Fig.  1h) (Petersen et al. 2021). In GAERS rats, we identified 13 PYR, 5 RSI, and 6 FSI neurons, while in Stargazer mice the distribution was similar, with 19 PYR, 5 RSI, and 4 FSI neurons. The electrophysiological properties of corresponding cell types were comparable across species (*P* > 0.19 for PYR spike width and τ; *P* = 0.26 for FSI τ; *P* = 1.0 for RSI width), supporting pooled analyses (Fig.  1h). Thus, the two datasets were sufficiently similar to examine ictal modulation at both population and cell-type levels.

**Figure 2:**
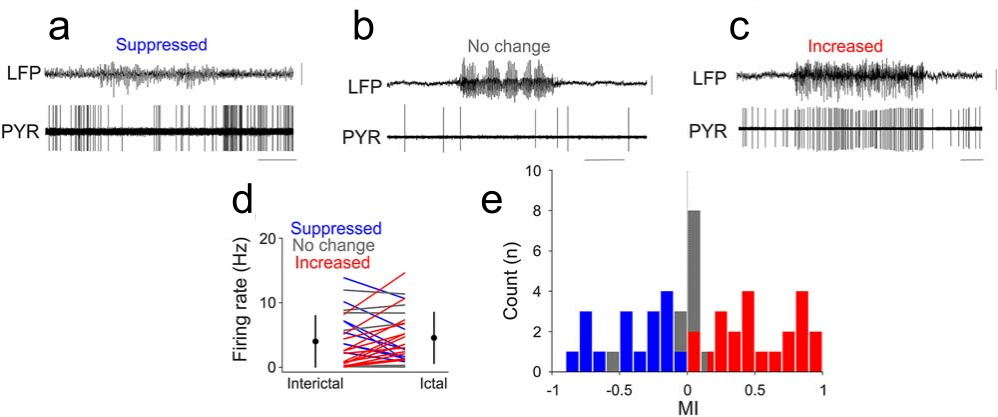
Ictal and interictal firing properties of neocortical pyramidal neurons. (a) Example simultaneous recording of M1 LFP and single unit recording of a pyramidal neuron of a stargazer mouse characterized by suppressed ictal firing activity. (b) Example simultaneous recording of S1BF LFP and single unit recording of a pyramidal neuron characterized by unaltered ictal firing activity. (c) Example simultaneous recording of S1BF LFP and single unit recording of a pyramidal neuron characterized by enhanced ictal firing activity. (d) Mean interictal and ictal firing rates of all pyramidal neurons recorded. Lines indicate individual neurons; circles indicate mean and standard deviation. Blue lines and bars indicate significant ictal suppression, gray lines and bars indicate no significant change, and red lines and bars indicate significant ictal increase in firing rates, respectively. (e) Distribution of modulation indexes (MI) in all recorded pyramidal neurons. Calibrations: 0.5 mV, 5 s.

**Figure 3:**
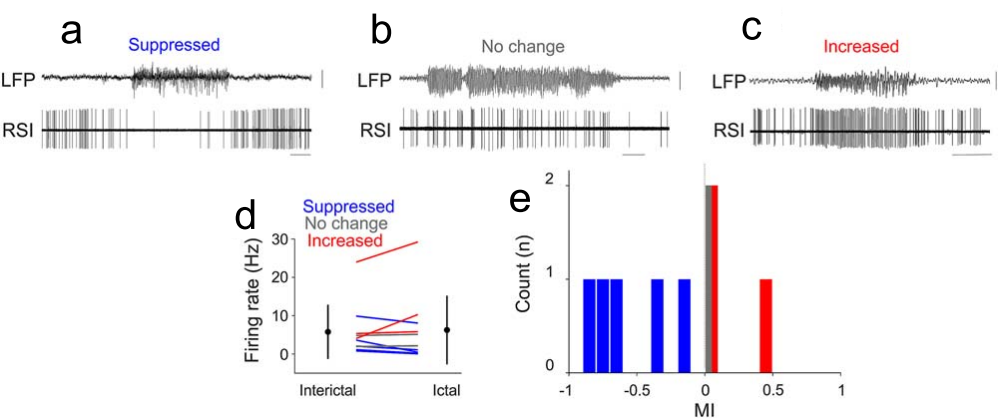
Ictal and interictal firing properties of neocortical regular spiking interneurons. (a) Example simultaneous recording of S1BF LFP and single unit recording of a regular spiking interneuron characterized by suppressed ictal firing activity. (b) Example simultaneous recording of S1BF LFP and single unit recording of a regular spiking interneuron characterized by unaltered ictal firing activity. (c) Example simultaneous recording of S1BF LFP and single unit recording of a regular spiking interneuron characterized by enhanced ictal firing activity. (d) Mean interictal and ictal firing rates of all RSIs recorded. Lines indicate individual neurons; circles indicate mean and standard deviation. Blue lines and bars indicate significant ictal suppression, gray lines and bars indicate no significant change and red lines and bars indicate significant ictal increase in firing rates, respectively. (e) Distribution of modulation indexes (MI) in all recorded RSIs. Calibrations: 0.5 mV, 5 s.

**Figure 4:**
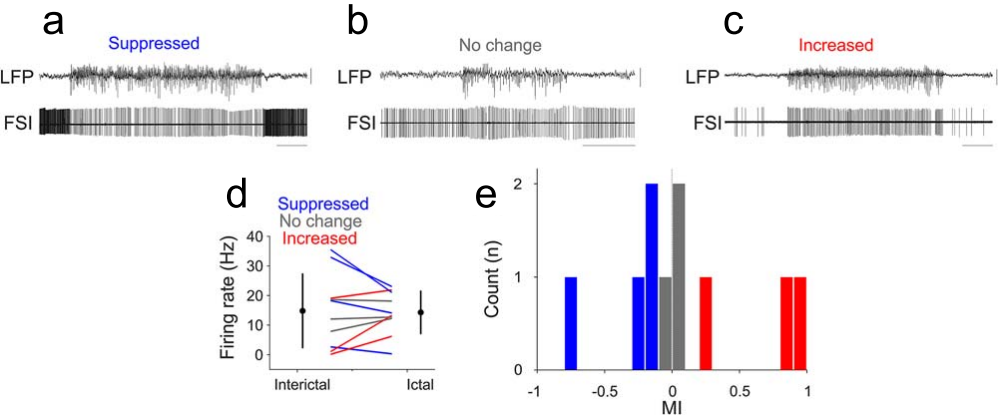
Ictal and interictal firing properties of neocortical fast-spiking interneurons. (a) Example simultaneous recording of S1BF LFP and single unit recording of a fast-spiking interneuron characterized by suppressed ictal firing activity. (b) Example simultaneous recording of S1BF LFP and single unit recording of a fast-spiking interneuron characterized by unaltered ictal firing activity. (c) Example simultaneous recording of S1BF LFP and single unit recording of a fast-spiking interneuron characterized by enhanced ictal firing activity. (d) Mean interictal and ictal firing rates of FSIs recorded. Lines indicate individual neurons; circles indicate mean and standard deviation. Blue lines and bars indicate significant ictal suppression, gray lines and bars indicate no significant change and red lines and bars indicate significant ictal increase in firing rates, respectively. (e) Distribution of modulation indexes (MI) in all recorded FSIs. Calibrations: 0.5 mV, 5 s.

Across all neurons, FSIs fired faster than PYR and RSI neurons during interictal periods (FSI: 14.82 ± 12.49 Hz; PYR: 5.88 ± 9.86 Hz; RSI: 5.76 ± 6.94 Hz; Kruskal–Wallis *P* = 0.0097), but during seizures mean firing rates converged across types (*P* = 0.70). Firing was temporally more regular in FSIs (ictal CV = 0.54 ± 0.77) than in PYR (1.09 ± 1.25) or RSI (1.74 ± 2.34; *P* = 0.046). Neither the MI nor the entrainment index (EI) differed significantly across cell types (*P* ≥ 0.70). Within each group, ictal rate modulation was heterogeneous. Among pyramidal neurons, 15 (48%) increased, 8 (26%) decreased, and 8 (26%) showed no significant change in firing rate (interictal 4.42 ± 5.55 Hz; ictal 4.71 ± 4.36 Hz; *P* = 0.20; Fig.  2). Among regular-spiking interneurons, 3 (30%) increased, 5 (50%) decreased, and 2 (20%) remained unchanged (interictal 5.76 ± 6.94 Hz; ictal 6.24 ± 8.82 Hz; *P* = 0.85; Fig.  3). Fast-spiking interneurons showed a similar pattern, with 3 (30%) increasing, 4 (40%) decreasing, and 3 (30%) showing no significant change (interictal 14.82 ± 12.49 Hz; ictal 14.31 ± 7.19 Hz; *P* = 1.0; Fig.  4). These proportions were comparable in GAERS and Stargazer datasets, indicating that heterogeneous modulation characterized all major cortical cell types independent of species or cortical area.

**Figure 5:**
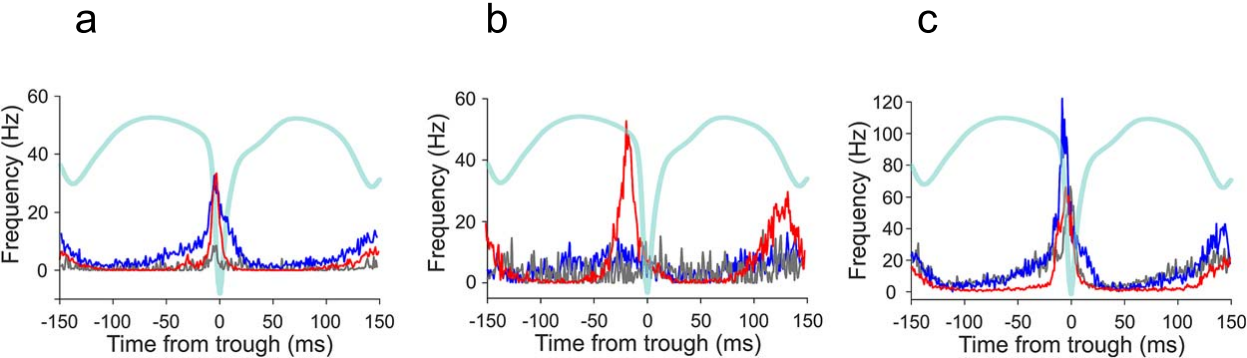
Spike–wave entrainment scales with ictal firing-rate modulation. Peri-event time histograms showing spike timing relative to individual SWD cycles for (a) pyramidal neurons, (b) regular-spiking interneurons, and (c) fast-spiking interneurons. Neurons are grouped by modulation subclass: Increased (red), suppressed (blue), and no change (gray). Traces represent mean firing rate aligned to the SWD cycle, with the averaged SWD waveform overlaid for reference.

To quantify neuronal coupling strength to the individual cycles of SWDs, we computed event-triggered PSTHs and derived modulation and phase-concentration metrics for all recorded neurons. Entrainment differed systematically across modulation subclasses. All recorded pyramidal neurons showed significant trough-locked modulation, but coupling strength differed markedly among sub-types (Fig.  5a). Spike–wave locking was strongest in neurons that increased their firing during seizures, with higher peak-to-baseline modulation (MI = 0.95) and greater phase concentration (vector strength = 0.59) compared to neurons with no change (MI = 0.78, VS = 0.20) or decreased firing (MI = 0.73, VS = 0.21). Peak responses in neurons with increased ictal firing reached 58 baseline SD units (z-peak), whereas neurons without ictal firing rate modulation and ictally suppressed neurons showed more moderate event-locked amplification (~9 to 10 SD). These results indicate that ictal recruitment strength is structured and differs across modulation-defined neuronal groups. In regular-spiking interneurons, a distinct pattern of ictal recruitment was observed (Fig.  5b). Across RSI subclasses, firing rates were modulated during seizures (MI ≈ 0.47 to 0.57), but phase concentration was generally weak, with low vector strength values (0.03 to 0.30) and modest peak amplification (z-peak ≈ 3 to 4). Fast-spiking interneurons also exhibited structured but heterogeneous spike–SWD coupling (Fig.  5c). FS neurons with ictal firing increases showed robust event-locked modulation (MI = 0.87) and large peak amplification (z-peak = 57.8), although their phase concentration was lower than that of pyramidal neurons (vector strength = 0.34). In contrast, non-modulated FS neurons showed weak and inconsistent coupling (MI = −0.14; vector strength = 0.09). Notably, FS neurons with ictal firing decreases displayed very large peak firing rates but minimal phase concentration (vector strength = 0.017), indicating that high firing rates do not necessarily imply precise temporal locking to spike–wave cycles. These findings demonstrate that neuronal recruitment during absence seizures is highly heterogeneous and dissociates firing magnitude from temporal precision. In contrast to other neuronal groups, RSI phase locking did not scale with firing increases; instead, neurons with reduced ictal firing exhibited slightly higher phase concentration. These results indicate that this neuronal group participates in ictal dynamics primarily through rate modulation rather than precise cycle-by-cycle entrainment.

We next examined the temporal structure of firing using two complementary metrics. The rhythmicity index (RI), defined as the ratio of the first side-peak to the mean peak in autocorrelations, captured cycle-to-cycle regularity, whereas the entrainment index (EI) quantified the fraction of spikes occurring during the LFP “spike” phase of spike-and-wave discharges (SWDs). RI values averaged 0.88 ± 0.58 for PYR, 1.25 ± 0.60 for RSI, and 0.77 ± 0.28 for FSI, with no significant group differences (*P* > 0.05, one-way ANOVA). RI did not correlate with either MI (r = 0.11, *P* = 0.61) or ictal firing rate. In contrast, EI was positively correlated with MI (r = 0.39, *P* = 0.0043; data not illustrated), indicating that neurons more strongly phase-locked to SWD cycles also exhibited greater firing-rate modulation. These analyses reveal that ictal modulation comprises two distinct but related dimensions: overall rate scaling (MI) and temporal alignment (EI). The coexistence of stable firing rank order and graded entrainment demonstrates that seizures reorganize, rather than randomize, cortical activity.

**Figure 6:**
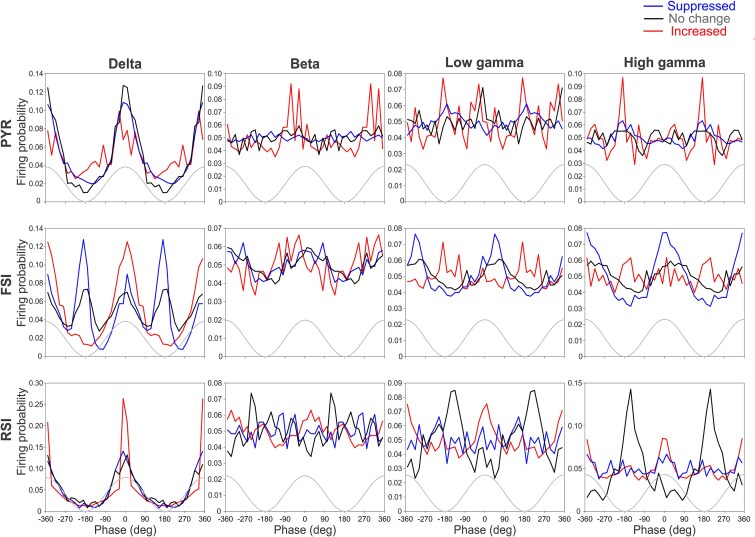
Interictal entrainment of neuronal firing to physiological oscillations across frequency bands and modulation subclasses. Phase distributions of spike timing relative to band-limited LFP oscillations during interictal periods are shown for pyramidal neurons (top row), fast-spiking interneurons (middle row), and regular-spiking interneurons (bottom row). Columns show coupling to delta (1 to 4 Hz), beta (13 to 30 Hz), low gamma (30 to 50 Hz), and high gamma (50 to 80 Hz) rhythms. Neurons are grouped according to their ictal firing-rate modulation: Increased (red), decreased (blue), and no change (gray). Spike phases were computed from the Hilbert-transformed LFP, and distributions are plotted over two consecutive cycles, with phase aligned such that 0° corresponds to the oscillation trough (range: −360° to +360°). The gray waveform indicates the phase of the underlying oscillation. These plots illustrate frequency- and cell-type–specific differences in interictal rhythmic coupling across modulation-defined neuronal subclasses, enabling comparison with ictal spike–wave entrainment.

To determine whether this temporal structure reflects pre-existing network dynamics, we analyzed the relationship between ictal firing-rate modulation and interictal rhythmic entrainment. Specifically, we quantified neuronal entrainment to physiological rhythms during interictal activity, including delta (2 to 4 Hz), beta (13 to 30 Hz), low gamma (30 to 50 Hz), and high gamma (50 to 80 Hz) bands (Fig.  6). For each frequency band, spike timing was referenced to the instantaneous phase of the oscillation obtained using Hilbert transform–based phase extraction, allowing quantification of phase preference and concentration across neurons. Neuronal coupling to interictal rhythms varied across frequency bands and cell types. In the delta band, most neurons exhibited strong phase coupling, with clear bimodal phase distributions observed particularly in fast-spiking interneurons, consistent with distinct subpopulations exhibiting different preferred phases. By contrast, higher-frequency bands showed weaker and more selective coupling patterns.

Across the population, coupling strength did not differ significantly across modulation subclasses in the delta, beta, low gamma, or high gamma bands (all *P* > 0.1), although beta-band coupling tended to be higher in neurons that increased firing during seizures than in non-modulated or suppressed neurons. At the level of continuous relationships, delta-band coupling was negatively correlated with modulation index across all neurons (ρ = −0.339, *P* = 0.035), whereas no significant band-specific correlations with modulation index were detected within individual cell classes. To determine whether interictal coupling contributes independently of baseline firing rate, we performed multivariate analyses. In these models, beta-band coupling remained a significant predictor of modulation index after accounting for interictal firing rate and cell type (*P* = 0.0061), whereas baseline interictal firing rate was not significant. By contrast, absolute modulation strength (|MI|) was primarily explained by baseline firing rate rather than rhythmic coupling. Interictal rhythmic coupling did not show a simple bivariate relationship with ictal spike–wave entrainment. However, in multivariate models controlling for baseline firing rate and cell type, low- and high-gamma coupling significantly predicted entrainment index (*P* = 0.0278 and *P* = 0.0137, respectively), indicating that physiological rhythmic embedding contributes differently to ictal firing-rate modulation and ictal temporal alignment. Together, these findings indicate that interictal rhythmic embedding is frequency- and cell-type–specific. Delta rhythms provide a broad temporal scaffold across neurons, beta-band coupling contributes selectively to ictal firing-rate modulation, and gamma-band coupling relates more closely to ictal spike–wave entrainment when cell type and baseline activity are taken into account.

**Figure 7:**
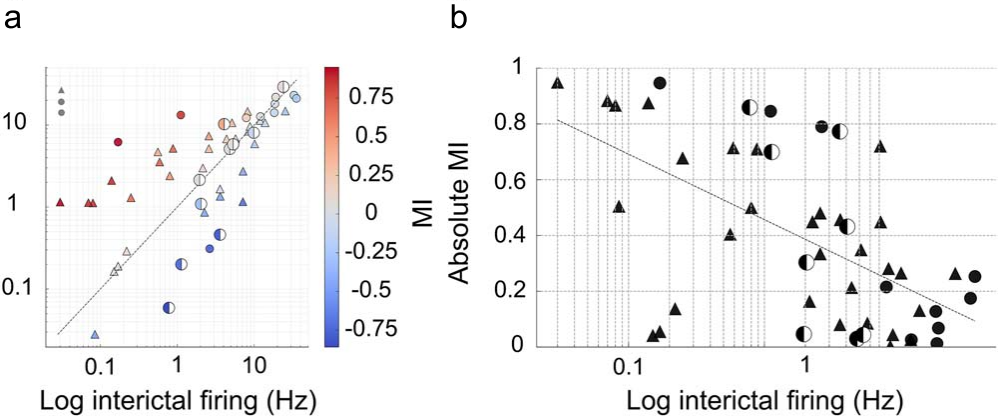
Firing rate modulation of neocortical neurons during absence seizures. (a) Scatter plot of interictal vs. ictal firing rates (log–log scale). Each point represents a single neuron, with symbol shape indicating cell type (▲ pyramidal neurons, ● fast-spiking interneurons, ◐ regular-spiking interneurons). Colors denote modulation index (MI), with warmer hues indicating ictal increases and cooler hues indicating decreases. The dashed unity line indicates equal ictal and interictal firing. (b) Relationship between baseline interictal firing rate and the absolute MI. Symbols and colors follow the same convention as in (a). The dashed line shows a log-linear regression fit, illustrating the negative correlation between baseline rate and modulation strength.

To place the observed heterogeneity and temporal structure into a population framework, we examined relationships between interictal and ictal firing across all neurons. Across the entire population, interictal and ictal firing rates were strongly correlated (Spearman ρ = 0.79, *P* = 1.6 × 10^−12^; Fig.  7a), indicating that neurons largely preserved their relative firing rank across brain states. Firing variability was likewise conserved, as interictal and ictal coefficients of variation were positively correlated (r = 0.67, *P* = 4.6 × 10^−8^; data not illustrated).

The magnitude of ictal modulation, quantified as the absolute modulation index (|MI|), was inversely related to baseline interictal firing rate (Spearman ρ = −0.60, *P* = 2.7 × 10^−6^; Fig.  7b), indicating that neurons with lower baseline firing rates tended to exhibit stronger ictal changes. Importantly, this relationship remained significant after accounting for cell type in a multivariate model (*P* < 0.0001 for baseline firing rate), demonstrating that reduced modulation in high-rate neurons cannot be explained solely by differences between pyramidal neurons and interneuron subclasses. When examined within individual cell classes, the negative relationship between baseline firing rate and ictal modulation was strongest and statistically significant within pyramidal neurons (ρ = −0.55, *P* = 0.0013), which constituted the largest and most heterogeneous population. Interneuron subclasses showed similar trends that did not reach significance, likely reflecting limited sample sizes.

## Discussion

Recordings were performed in different cortical regions in GAERS rats and Stargazer mice to target the presumed cortical sites of seizure initiation in each model. In GAERS rats, absence seizures consistently originate in the S1 barrel cortex, whereas in Stargazer mice available evidence points to a more frontal cortical origin. Targeting these regions allowed us to assess neuronal activity at sites of maximal ictal engagement. Thus, the observed heterogeneity in neuronal firing cannot be attributed to recordings made in cortical areas remote from seizure initiation but instead reflects intrinsic diversity of neuronal responses within the seizure-generating network itself. Building on these findings, we propose that the observed structured heterogeneity reflects a scalable reconfiguration of pre-existing cortical network dynamics rather than a uniform increase in excitability. Despite substantial variability in individual firing rates, neurons preserved their relative firing rank and variability across interictal and ictal states, indicating that seizures do not impose a uniform excitatory drive but instead modulate pre-existing network organization. This challenges the classical view of generalized cortical hypersynchrony and supports an alternative framework in which pathological oscillations reflect reorganized, intrinsically structured network activity.

Previous studies performed under anesthesia have reported highly stereotyped rhythmic bursting of cortical, thalamic, and striatal neurons during absence seizures (Crunelli et al. 2020). In contrast, recent work in awake behaving animals has shown greater variability and weaker entrainment of neuronal firing (McCafferty et al. 2018, 2023; Meyer et al. 2018; Miyamoto et al. 2019). Our results extend these observations by demonstrating that ictal heterogeneity is not limited to population averages but is a fundamental property of individual cortical neurons, encompassing both excitatory and inhibitory cell types in the cortical initiation zone of SWDs. This suggests that the homogeneity observed in anesthetized preparations likely reflects the artificial synchrony induced by reduced brain-state dynamics rather than a defining feature of absence seizures themselves.

The preservation of firing rank order together with graded entrainment mirrors human findings in which conditional-inference and maximum-entropy models attribute most fine synchrony to nonstationary rate modulation and local network activation, supporting a scaled, structured modulation rather than uniform hypersynchrony (Keller et al. 2010; Truccolo et al. 2011). Diversity at the single-unit level is present during both interictal discharges and ictal epochs in humans, with pre-event modulations localized near the focus and heterogeneous fast-component responses, a finding that parallels our awake cortical results (Keller et al. 2010; Truccolo et al. 2011).

By resolving ictal recruitment at both the population and sub-cycle temporal scales, our data demonstrate that absence seizures do not impose a uniform mode of cortical engagement. Instead, neuronal participation is structured and strongly cell-type dependent. Neurons that increased their firing during seizures exhibited the most precise spike–wave coupling, with strong phase concentration and narrow peri-trough firing, particularly among pyramidal cells. In contrast, fast-spiking interneurons displayed a marked dissociation between firing magnitude and temporal precision: some interneurons fired intensely without consistent phase locking, indicating that high firing rates do not necessarily reflect synchronous recruitment. Regular-spiking interneurons showed a third pattern, characterized by moderate firing modulation but weak phase concentration, suggesting that their ictal participation is dominated by rate changes rather than cycle-by-cycle entrainment. Together, these findings argue against a generalized hyperexcitability or uniform synchrony model of absence seizures and instead support the view that ictal activity represents a scalable reconfiguration of pre-existing cortical dynamics, in which distinct neuronal classes preserve their characteristic temporal roles.

To further examine whether this structured heterogeneity reflects pre-existing network dynamics, we analyzed neuronal entrainment to physiological rhythms during interictal periods. Interictal phase coupling revealed a clear frequency-dependent organization of neuronal activity. Delta-band coupling was widespread across neurons, with bimodal phase distributions in fast-spiking interneurons indicating the presence of distinct functional subpopulations. This suggests that slow cortical rhythms provide a global temporal scaffold within which neuronal activity is organized during interictal periods. In contrast, higher-frequency bands exhibited more selective relationships to ictal dynamics. Although beta-band coupling did not differ significantly across modulation subclasses at the population level, it emerged as a significant predictor of ictal firing-rate modulation in multivariate analyses, independent of baseline firing rate and cell type. This indicates that the degree to which neurons are embedded in ongoing beta-frequency activity is associated with the magnitude of ictal firing-rate modulation during seizures. By comparison, gamma-band coupling showed a different relationship to ictal dynamics. While it did not predict firing-rate modulation, both low- and high-gamma coupling were associated with ictal spike–wave entrainment in multivariate models, suggesting that physiological high-frequency activity is associated with ictal spike–wave entrainment than to overall rate changes. Together, these findings indicate that distinct components of interictal rhythmic activity differentially map onto ictal dynamics, supporting the view that seizures reorganize, rather than override, pre-existing cortical network dynamics across multiple temporal scales.

The coexistence of heterogeneous cellular firing with stereotypical EEG SWDs implies that cortical networks employ normalization mechanisms to stabilize overall output despite diverse single-cell responses. In this framework, seizures maintain consistent macroscopic patterns by scaling the activity of individual neurons relative to the collective network drive. Similar divisive normalization processes have been described in sensory and motor cortices, where neuronal responses are proportional to the pooled activity of neighboring neurons (Carandini and Heeger 2011). The preserved firing rank order observed here is consistent with such a normalization principle, suggesting that absence seizures modulate the amplitude, but not the structure, of cortical activity. Whether the preservation of firing-rate rank order across states is accompanied by conserved temporal firing sequences between neuronal subtypes remains an important open question that will require simultaneous recordings from larger neuronal populations.

Several cellular and synaptic mechanisms could underlie this normalization. Conductance increases—particularly those mediated by GABAA receptor–driven shunting inhibition—can scale membrane potential responses without altering net synaptic current (Borg-Graham et al. 1998). Balanced elevations of excitatory and inhibitory conductances can therefore adjust neuronal gain divisively (Silver 2010), enabling neurons to maintain proportional firing across brain states. Stochastic fluctuations in synaptic input from connected neurons may further induce dynamic conductance changes that reinforce this divisive scaling (Chance et al. 2002). Together, these mechanisms can yield heterogeneous single-cell firing while maintaining stable network-level activity, reconciling cellular variability with the stereotyped EEG manifestations of SWDs.

Beyond local circuit mechanisms, broader network interactions likely contribute to the observed dynamics. Infragranular layers of the barrel cortex receive substantial non-sensory input from motor regions, both directly and via the posterior-medial thalamic nucleus (Miyashita et al. 1994; Deschênes et al. 1998). Such projections could introduce motor-related modulation of sensory cortical gain, a form of dynamic normalization that adjusts responsiveness based on behavioral context (Seki et al. 2003; Nguyen and Kleinfeld 2005). Internal, non-sensory afferents to thalamic nuclei can also depress thalamocortical synapses (Castro-Alamancos and Oldford 2002), further shaping synchronization patterns and promoting multicluster dynamics, in which subsets of neurons synchronize without complete population entrainment (Amritkar and Rangarajan 2009). These mechanisms could collectively explain how a heterogeneous cortical network produces the characteristically regular SWD pattern in the EEG.

A key limitation of the present study is the relatively small number of recorded neurons, reflecting the technical challenges of achieving long-term stability for juxtacellular recordings in awake animals while capturing spontaneous seizures. Nevertheless, this approach ensures that the recorded signals originate unambiguously from single neurons, which is critical when assessing neuronal heterogeneity. A further limitation is that recordings were obtained from adult rodents, whereas absence epilepsy in humans is primarily a pediatric disorder that often remits by late adolescence. This reflects a general constraint of animal models, as invasive cellular recordings during seizures are not feasible in human patients. Despite this difference, GAERS rats and Stargazer mice reproduce the defining electrographic features of absence epilepsy and provide unique access to the neuronal mechanisms underlying spike-and-wave discharges. Our results therefore speak to general principles of ictal network organization rather than age-specific clinical manifestations.

Together, our findings indicate that seizures do not erase neuronal identity but modulate it in a structured and predictable manner. Neurons with distinct intrinsic and synaptic properties preserve their firing relationships across brain states, implying that the seizure state is not a chaotic departure from normal activity but a scaled reconfiguration of the pre-existing cortical network. Viewing seizures as an emergent, graded reorganization of ongoing activity rather than as a uniform cortical storm reframes our understanding of epileptic dynamics and offers new avenues for developing predictive and cell-type–specific interventions.
